# Cognitive Ageing in Great Britain in the New Century: Cohort Differences in Episodic Memory

**DOI:** 10.1371/journal.pone.0144907

**Published:** 2015-12-29

**Authors:** Gindo Tampubolon

**Affiliations:** Cathie Marsh Institute for Social Research, University of Manchester, Manchester, United Kingdom; University of Lethbridge, CANADA

## Abstract

**Background:**

Dementias in high income countries are set to be the third major burden of disease even as older people are increasingly required to think for themselves how to provide for their lives in retirement. Meanwhile the period of older age continues to extend with increase in life expectancy. This challenge demands an understanding of how cognition changes over an extended period in later life. But studying cognitive ageing in the population faces a difficulty from the fact that older respondents are liable to leave (attrite) before study completion. This study tested three hypotheses: trajectories of cognitive ageing in Britain show an improvement beyond the age of 50; and they are lifted by secular improvement in cognition across cohorts; lastly they are susceptible to distortion due to attrition.

**Methods and Findings:**

Using the English Longitudinal Study of Ageing, this paper studied trajectories of episodic memory of Britons aged 50–89 from 2002 to 2013 (*N* = 5931). Using joint models the analysis found that levels of episodic memory follow a curvilinear shape, not a steady decline, in later life. The findings also revealed secular improvement in cognitive ageing such that as a cohort is being replaced episodic memory levels in the population improve. The analysis lastly demonstrated that failure to simultaneously model attrition can produce distorted pictures of cognitive ageing.

**Conclusion:**

Old age in this century is not necessarily a period dominated by cognitive decline. In identifying behavioural factors associated with better cognitive ageing, such as social connections of traditional and online kinds, the paper raises possibilities of mustering an adequate response to the cognition challenge.

## Introduction

In over a decade all dementias including Alzheimer’s disease are projected to rank as the third largest burden of disease in high income countries. Of more than 112,000 disability-adjusted life years lost to all diseases, nearly 6% or 6545 years are lost to dementias in ageing populations [1]. This loss makes understanding cognitive change in later life crucial since cognitive decline can precede dementias by a long period [2]. A study crystallised this challenge to public health by calling for more systematic research on how cognitive function changes and declines in older people [3].

With the projected rise in the proportion of older people in these countries, the older populations are increasingly asked to share the fiscal burden of caring for later life [4–8]. Many middle aged and older people are encouraged to consider a variety of choices for providing for old age [9, 10]. Making this choice requires not only accurate information from the outside but also well functioning cognition in the individual. Thus individual trajectories of cognitive function in old age need to be understood in order to achieve an economically efficient individual choice in old age provision.

For policy purpose the ageing populations in high income countries have often been simply charaterised and compared in terms of old age dependency ratio. But it is increasingly apparent that growing old in these different countries entails different experiences. Such a comparative exercise can only benefit from incorporating the varying levels of cognitive function in the older populations [11]. After all, identical ratios of old to working age people in two countries would nonetheless prompt a pause if the levels of cognitive function of older people in the two countries differed markedly.

Cognition has been the subject of excellent investigations in the past, and one of the results showed that in the general population over recent decades it has been secularly improving, an observation known as the ‘Flynn effect’[12–17]. But for the specific population of older people, this claim of an improvement has been contested [10, 18]. The Flynn effect largely refers to the levels in the general population, somewhat distinct from the levels among older people. This possibility has not received the same currency in the psychological literature [19]. So from public health, psychology and policy perspectives, a study of cognitive change in older people in recent years is needed urgently.

Recent works have responded to this challenge. Cross-sectional studies make up a majority of this body of work, and they tend also to be community or volunteer sample rather than national sample studies. For example, a cross-sectional study analysed people aged 50 years and over in 2002 in the English Longitudinal Study of Ageing (ELSA) wave 1 to examine socio-economic differences in cognitive function [20]. Using the same longitudinal sample enhanced with wave 2, a different study followed by presenting yet another cross-sectional study of cognitive function and of self-reported change in cognition [21]. Using mixed models, an investigation in the Whitehall II study focused on decline in cognitive function in a community sample of civil servants in London (Whitehall being the metonym for the cream of the UK civil service)[22]. Notably, the study suffered from 20% sample attrition in 10 years. The authors showed that cross-sectional results, compared to longitudinal ones, overestimate the speed of cognitive decline.

In contrast to the Whitehall study, in substantially African-American neighbourhoods in Chicago, another longitudinal study followed older people for over 12 years [23]. As has been intimated, attrition is an acute problem rarely addressed in longitudinal study including the Whitehall study above, and a method to deal with this is demonstrated in the Chicago study. The authors show that ignoring attrition, compared to incorporating it, underestimates the contribution of risk factors for cognitive decline. Neither the Whitehall civil servants nor the Chicago neighbourhood residents are representative of the populations on both sides of the Atlantic. Beyond the Pacific, attrition bias is also found in a longitudinal study of cognitive ageing in south Australia [24].

Because this active research has mapped out a broad landscape of cognitive function changes in older people in high income countries, we can see major gaps that need addressing. First, studies of longitudinal change in cognitive function are few, especially those over an extended period for a nationally representative sample. Exceptionally, McArdle and colleagues studied the American sample from 1992 to 2004 [13] while Gale and colleagues used the British sample (ELSA) from 2002 to 2009 [17]; however the last study ignored practice effect and attrition (more on this below). Studies also tend to leave out a large swathe of the population, limiting the scope for generalisation. For instance, the sample of high ranking civil servants in the capital necessarily left out many more others who live and work up and down the country. Similarly Chicago as the site of one study above has been noted as not necessarily representative of the US [25]. Equally, down under in south Australia, many older Australians outside Adelaide may trace different trajectories of cognitive function in old age. Thus given the increasing proportion of older people in Britain, a nationally representative picture of cognitive ageing is needed.

Another major gap is symptomatic of an aspect of the first one (extended longitudinal change), and this has to do with attrition in longitudinal studies. Samples in longitudinal ageing studies, whether nationally representative or not, tend to suffer from systematic attrition, since older people left the studies not at random. From the limited studies examining cognitive change longitudinally, even fewer consider attrition, despite its being common. This gap is particularly salient for ELSA, as recent works have made clear that the scale of the problem with ELSA deserves considerable care [26, 27]. Although ELSA has been used to draw trajectories of quality of life while accounting for attrition [27], the same has not been done for trajectories of cognitive function which naturally suffer from the same attrition. In comparison, trajectories of cognitive function jointly modelled with attrition have been drawn for residents of Adelaide, estimating a handful of risk factors [24]. Attrition must not be ignored if we are to obtain clear pictures of cognitive ageing.

Some of these studies also tended to examine a limited set of risk factors beyond sex and age [13]. A careful investigation needs to consider broad social determinants such as education, occupation, wealth, social network, as well as comorbidities, and not least, behavioural life styles such as physical exercise and alcohol consumption [22–24]. An extensive set of risk factors should be examined to gain robust trajectories of cognitive function in older age.

While the above gaps of selective samples, limited factors, and attrition threaten any ageing study say on physical functions, a study on cognitive ageing nevertheless poses an additional challenge that is seldom met. Repeated measurement of cognitive function creates a practice effect that can mask true changes in cognitive function [14, 15, 19, 28, 29].

To deal with this practice effect, a consensus is emerging with the help of three categories of practical approach [30]. Only the first of these approaches, by including practice indicator(s) as a fixed [14] or random effect(s) [13], is readily applicable to an ongoing longitudinal ageing study as opposed to the other two approaches, which require redesign of or changes to the administration of the longitudinal ageing study. At any rate, practice effect has rarely been examined in the national sample of older Britons.

This study therefore aims to characterise the shape of cognitive function trajectories in Britain today. To focus our effort three hypotheses are tested. First, trajectories of cognitive function in British people aged 50 and older are curvilinear, showing not only decline but also increase in episodic memory. Second, the trajectories are lifted by a secular improvement in cognition across cohorts. Lastly, the trajectories are susceptible to distortion due to attrition masking real change experienced by older people. For these purposes ELSA is used, an ongoing longitudinal study of people aged 50 and over from 2002 to 2013.

The analysis found that cognitive function traced a curvilinear shape that peaks in the early 60s. This contradicts most studies which put the peak variously in the 20s, 30s and 40s [22, 29, 31]. This finding practically means that, a decade into ELSA which covered 2002 to 2013, for a large section of the British older population the peak in cognition is just coming. The analysis also showed extensive social inequalities in trajectories of cognitive ageing. In particular, wealth, class and education are strongly associated with the maintenance of cognitive function. Importantly, the analysis also showed that using common models (growth curve or random coefficients or mixed models which assumed attrition at random) modified the trajectories of cognitive function and the contributions of the risk factors.

## Materials and Methods: The English Longitudinal Study of Ageing, 2002–2013

The English Longitudinal Study of Ageing (ELSA) is the primary resource for a nationally-representative ageing study of the English population aged 50 years and older, started in 2002 and subsequent waves follow biennially. The study was funded by a consortium of UK Government Departments and the US National Institute of Aging; the data are freely available from the UK Data Archive www.data-archive.ac.uk as study number 5050. ELSA is a multidisciplinary study that contains a broad range of information collected repeatedly, including details on health, economic and social circumstances, retirement or transitions throughout later life. Every four years, a nurse assessment is given to collect biomedical information, including from blood, saliva and hair samples. More details of the study are given elsewhere [32–37].

ELSA, like many other longitudinal ageing studies, suffers from attrition. The extent of the attrition problem in ELSA and its US sister study has been shown before [26]; and how attrition in ELSA masked improvement in the life quality of older people has also been demonstrated [27]. Following a cognitive ageing study using Australian data [24], and a recent quality of life study using the same ELSA data, attrition is handled using a joint model [27].

### Ethics review

Ethical approval for all the ELSA waves was granted from the National Research and Ethics Committee of the UK National Health Service www.nres.nhs.uk. The University of Manchester’s institutional review board has exempted this study since it used publicly available anonymised secondary data for research.

### Dependent variable: episodic memory

Because cognitive functioning is a multivariate concept, no single cognitive function measure has commanded a consensus in the literature; thus episodic memory, the sum of delayed and immediate recall, is used. This measure has been known to have good construct validity and consistency [38, 39]. Importantly, episodic memory is also known to relate to every-day activities of older people as well as to their critical decisions such as on pension provision [10].

### Independent variables

Information from respondents aged 50 to 89 was used, since age is capped at 90 in ELSA. Demographic covariates include sex (Female), age and squared-age, to capture possible curvilinear trajectories [14, 15, 40].

Cohorts are birth groups marked by socio-historical events to make them comparable to the US sister study. The four cohorts (requiring three cohort indicators) are pre-Depression cohort (born before 1930, omitted as the reference), Depression era cohort (1931–1938), War cohort (1939–1945) and post-War cohort (born after 1946). This is more refined than the three cohorts used in the ELSA report [9, 20, 32]. In sensitivity analyses, instead of socio-historical cohorts, decadal calendar cohorts were tested, distinguishing cohort members in their 50s (born in 1943 to 1952), 60s, 70s and 80s. No substantial difference is found (see S1 Table).

Cognitive functions are known to be affected by physical functions. Thus all three dimensions of physical functions available in ELSA are used as a sum score of physical function, including (instrumental) activities of daily living, mobility and muscle functions [3].

Cognitive functions, like other health functions, are also shaped by social determinants of health [41, 42]. These determinants include occupational class (three categories: managerial, intermediate and routine manual class as reference; National Statistics Socio-Economic Classification [43]), wealth tertiles (top, middle and bottom as reference), marital status (married/cohabiting, divorced or separated and single as reference) [13], and education (at least some college, high school and less than high school as reference).

Education level in particular has been repeatedly shown to affect cognitive functioning in older people, either directly through the provision of cognitive reserve or indirectly through the occupational route [13, 44]. On this route, it is suggested that higher levels of education allow educated people to attain good jobs which provide cognitively stimulating tasks. These in turn enhance cognitive functioning well into late life.

It is not only the major social determinants such as wealth and occupation that can affect cognitive ageing. Broader social factors such as social networks can also influence cognitive functioning of older people through stimuli encountered in social interactions of various kinds, including traditional and online social interactions. A score is included for the frequency of social interactions with friends and family either in face-to-face meeting, by phone or by emails.

Since it is widely known that risk factors for cardiovascular disease (CVD) may be implicated in cognitive decline [45], it is advisable to also include these risk factors or cardiovascular disease status. In this connection, since comorbidities generally increase with age (not only CVD and cognitive problems), robust trajectories of cognitive ageing need to consider broader comorbidities as well. A series of indicators about chronic conditions are thus included, covering diabetes, cancer, CVD, arthritis and depression measured using CES-D score [17].

Behavioural risk factors known to be effective in cross-sectional studies include smoking (current smoker and not current smoker as reference), drinking (days in a week having a drink) and physical exercise (rigorous, moderate physical exercise and less as reference) [24].

Only those with complete information are retained in the following analysis. Differences between the analytic and excluded samples are tested using *t* test for continuous variable and *χ* ^2^ test for categorical variables as appropriate. The excluded sample tend to be older (69.9 versus 67.3 years; *p* < 0.001), and poorer (mean wealth £65229 versus £67805, *p* < 0.001). Additionally the proportion of women in the excluded sample is lower than that of women in the analytic sample (0.46 versus 0.54; *p* < 0.001), the proportion of managerial occupational class in the excluded sample is lower than that of managerial class in the analytic sample (0.25 versus 0.34, *p* < 0.001), and the proportion of degree-educated adults in the excluded sample is lower than that of degree-educated adults in the analytic sample (0.21 versus 0.29, *p* < 0.001). Therefore these variables are always included in the analysis.

#### Statistical analysis

Longitudinal cognitive ageing studies face two major empirical issues: practice effect in cognitive testing and attrition. Following many excellent analyses of cognitive ageing in Britain and the US [13–15] we first used maximum likelihood estimator of growth curve model with a missing at random assumption; this is variously known as random coefficients or mixed models. They are often used since they give consistent estimates if attritions are missing at random. This means that, given the observed history of covariates and dependent variables, those who left differ from those who stayed only in a random fashion [14, 15].

Episodic memory changes were modelled using a sequence of three models. In all models, practice effect was estimated by entering an indicator of repeat testing following the literature [13–15, 46, 47]. This remains the first choice (or the first approach in Salthouse’s categories [30]) for an ongoing longitudinal ageing study such as ELSA.

First, an initial model included age, squared age, sex, social determinants, chronic conditions and behavioural risk factors (*baseline model*); then cohort indicators were included in the *cohort model*.

We argued above that a joint model of cognition and attrition is needed [14, 24, 27]. Following a recent study which analysed this ELSA sample using joint models [27], we used joint models where the random effects (*h*(.) below) influence both episodic memory, *y*, and attrition, *t*; given these, episodic memory and attrition are independent. One part of the joint model is made up of the growth curve model (*f*(.)); the other part is a survival model (*g*(.)) with sex, age polynomial of degree three and the random intercepts from part one. The likelihood is [24, 27]

$$\begin{array}{c} L=\int f\left(y_{i}|b_{i},x_{i}\right)g\left(t_{i}|b_{i},x_{i}\right)h\left(b_{i}\right){\text{db}}_{i}. \end{array}$$ (1)

Joint models have been found to give robust estimates of cognitive ageing in Australia [24]. Joint models with similar specification have been successfully applied to this sample in explaining older Britons’ quality of life [27]. The robustness of the *cohort model* to attrition can be readily assessed by comparing its results with that of the *joint model* [14].

The three models (baseline, cohort and joint models) were put together in a table with three panes, with each model accompanied by *R* ^2^ fit statistics. All analyses are done in Latent Gold syntax 5 [48].

#### Sensitivity analysis

To facilitate future comparison, instead of socio-historical grouping, the sample has been grouped into four decadal cohorts. The results with this alternative grouping were given in S1 Table. No substantial difference appears that warrants modification of the following results.

## Results

The analytic sample (Table 1, *N* = 5931) with complete information on covariates is made up of more women (54%) than men and of more recent cohorts (31% of the most recent one compared to 19% of the earliest one). There is a graded increase in the levels of episodic memory across cohorts, such that on average the Post-War cohort can recall about four more words than the Pre-Depression era cohort. Social gradient in cognitive functions is evident along the three major socioeconomic statuses: education, social class and wealth. For instance, there is a monotone increase in the average cognitive functions (episodic memory) along increasing wealth tertiles. Lifestyle behaviours including drinking and physical exercise do not have a consistent pattern [49]. These bivariate associations, no doubt subject to confounding, were examined further using growth curve and joint models for episodic memory.

**Table 1:** Some descriptives of the analytic sample (ELSA 2002–2013).

Variable	Episodic memory	
	Mean, 9.97	Std. dev., 3.62
Sex		
Male (45%)	9.7	3.5
Female (54%)	10.2	3.7
Cohort		
Pre-Depression (19%)	7.5	3.5
Depression era (24%)	9.4	3.4
War (24%)	10.5	3.3
Post-War (31%)	11.5	3.2
Marital status		
Not in union (31%)	9.2	3.8
Married-cohab (68%)	10.3	3.5
Ethnicity		
Other (2%)	8.3	3.9
White (97%)	10.0	3.6
Social Class		
Managerial (34%)	10.9	3.5
Intermediate (23%)	10.4	3.5
Routine else (42%)	9.0	3.5
Education		
< college (70%)	9.4	3.6
SomeCollege (29%)	11.3	3.4
Wealth tertiles		
Bottom (31%)	9.1	3.7
Middle (34%)	9.8	3.6
Top (34%)	10.9	3.4
Hypertension		
No (56%)	10.3	3.6
Yes (43%)	9.6	3.6
Diabetes		
No (90%)	10.1	3.6
Yes (9%)	8.9	3.6
Cancer		
No (91%)	10.0	3.6
Yes (8%)	9.9	3.7
Heart condition		
No (80%)	10.1	3.6
Yes (19%)	9.3	3.8
Stroke		
No (95%)	10.1	3.6
Yes (4%)	8.0	3.7
Arthritis		
No (62%)	10.2	3.6
Yes (37%)	9.6	3.6
Smoker		
0 (36%)	10.2	3.6
1 (63%)	9.8	3.6
Drink 5–7 days p.week		
No (11%)	8.7	3.7
Yes (88%)	10.3	3.5
Moderate exercise		
0 (25%)	8.5	3.8
1 (74%)	10.5	3.4
Vigorous exercise		
0 (72%)	9.6	3.7
1 (27%)	10.9	3.3

**Fig 1:**
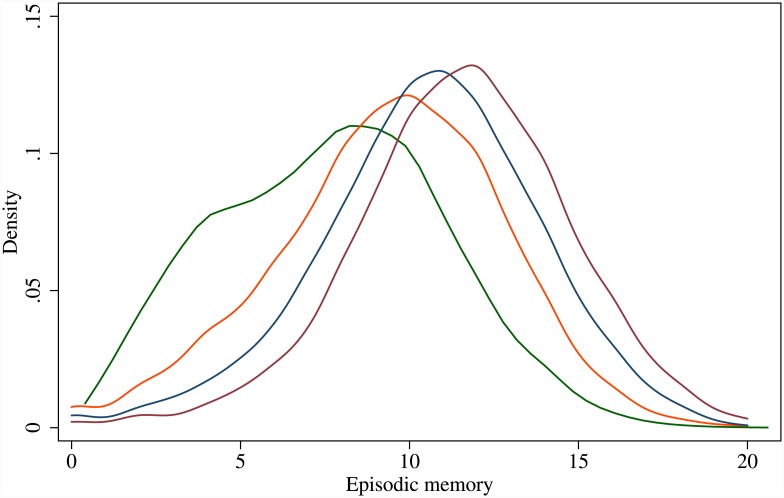
Empirical densities of episodic memory for four cohorts: Pre-Depression (left-most), Depression, War and Post-War (right-most) cohorts (Source: ELSA 2002–2013).

Empirical densities of episodic memory for the four cohorts are shown in Fig 1. This figure shows that recent cohorts displayed higher levels of episodic memory (right-shift). These shifts impress the need to include secular improvement across cohorts in explaining individual trajectories of cognitive ageing.

**Fig 2:**
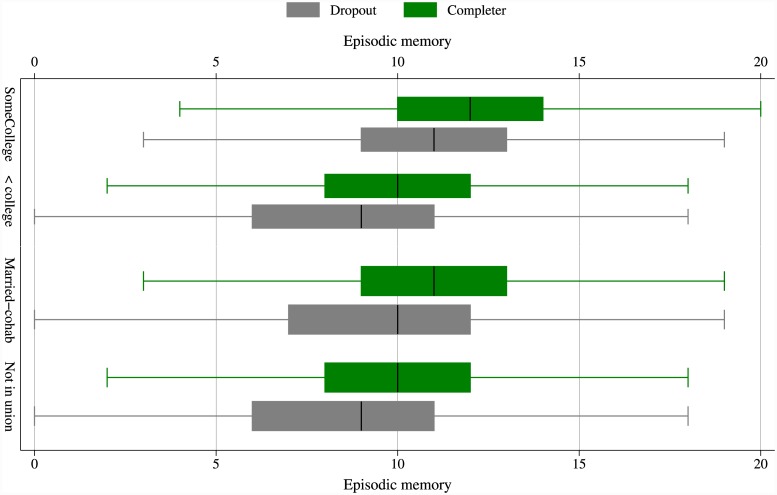
Box plots of episodic memory by education attainment and attrition status as well as by marital status and attrition status. (Source: ELSA 2002–2013).

If Fig 1 motivated cohort effects, the next figure illustrates attrition effect. Fig 2 shows evidence of how, even in 2002, the levels of episodic memory differ not only across social groups but also across attrition status. Married or cohabiting couples (compared to those not in union) tend to have higher episodic memory; but even more importantly, those who completed the course of study also have higher levels of episodic memory. This difference between those who completed and those who dropped out is apparent in both categories of marital status. Similarly with regards to education categories, those with college education have higher levels of episodic memory compared to those without, and within each level of educational attainment those who completed the study have higher levels of episodic memory, even at baseline. This raises the concern about attrition when explaining individual trajectories of cognitive ageing.

The *baseline model* for episodic memory includes curvilinear age effects, social determinants, chronic conditions and behavioural risk factors. The results (Table 2 left pane) showed that trajectories of episodic memory in later life have a curvilinear shape (age 0.4325, *p* < 0.001; age^2^ −0.0040, *p* < 0.001). With these coefficients the peak is reached at age 54.1 year.

**Table 2:** Episodic memory in later life (ELSA 2002–2013).

Predictor	Baseline	Cohort	Joint
Constant	−3.5811 ± 0.8142^†^	−9.1501 ± 0.8799^†^	−9.7657 ± 0.8513^†^
Age	0.4325 ± 0.0242^†^	0.4890 ± 0.0263^†^	0.5220 ± 0.0253^†^
Age^2^	−0.0040 ± 0.0002^†^	−0.0038 ± 0.0002^†^	−0.0042 ± 0.0002^†^
Depression	.	0.9052 ± 0.0782^†^	0.7867 ± 0.0782^†^
War	.	1.7263 ± 0.0885^†^	1.5513 ± 0.0879^†^
Post-War	.	2.6917 ± 0.0965^†^	2.4901 ± 0.0953^†^
Female	0.7314 ± 0.0472^†^	0.7195 ± 0.0466^†^	0.7020 ± 0.0463^†^
Intermediate	0.9673 ± 0.0585^†^	0.9521 ± 0.0576^†^	0.9842 ± 0.0575^†^
Managerial	1.1541 ± 0.0553^†^	1.0970 ± 0.0546^†^	1.1409 ± 0.0544^†^
Married/cohab.	−0.0133 ± 0.0440^†^	−0.0340 ± 0.0435	0.0044 ± 0.0423
College	1.0402 ± 0.0548^†^	0.0548 ± 0.0541^†^	0.9237 ± 0.0541^†^
Middle tertile	−0.1592 ± 0.0373^†^	0.1773 ± 0.0370^†^	0.1992 ± 0.0353^†^
Top tertile	0.3210 ± 0.0428^†^	0.3571 ± 0.0424^†^	0.3748 ± 0.0410^†^
Ethnic minority	−1.3915 ± 0.1393	−1.4193 ± 0.1374^†^	−1.5837 ± 0.1294^†^
Social connect	0.0177 ± 0.0184	0.0815 ± 0.0184^†^	0.0977 ± 0.0179^†^
Physical problem	−0.0448 ± 0.0063^†^	−0.0380 ± 0.0062^†^	−0.0435 ± 0.0059^†^
Hypertensives	−0.0121 ± 0.0384	−0.0670 ± 0.0380	−0.0610 ± 0.0373
Diabetes	−0.0913 ± 0.0631	−0.1705 ± 0.0624^†^	−0.2168 ± 0.0605^†^
Cancer	0.1962 ± 0.0642^†^	0.0941 ± 0.0636	0.1174 ± 0.0623
CVD	0.0701 ± 0.0477	0.0286 ± 0.0472	0.0344 ± 0.0461
Stroke	−0.6070 ± 0.0900^†^	−0.6126 ± 0.0891^†^	−0.6445 ± 0.0848^†^
Arthritis	0.3076 ± 0.0407^†^	0.2086 ± 0.0403^†^	0.2088 ± 0.0396^†^
Smoker	−0.0281 ± 0.0440^†^	−0.0467 ± 0.0434	−0.0504 ± 0.0431
Drink regularly	0.4432 ± 0.0513^†^	0.4675 ± 0.0507^†^	0.3455 ± 0.0491^†^
Exercise mod.	0.2576 ± 0.0351^†^	0.2584 ± 0.0347^†^	0.3092 ± 0.0329^†^
Exercise vig.	0.1150 ± 0.0328^†^	0.1040 ± 0.0325^†^	0.1110 ± 0.0317^†^
CESD	−0.0851 ± 0.0083^†^	−0.0772 ± 0.0083^†^	−0.0731 ± 0.0079^†^
Practice	0.9039 ± 0.0722^†^	0.6367 ± 0.0720^†^	0.5859 ± 0.0713^†^
Within-person *σ*	0.0379 ± 0.0017^†^	0.0370 ± 0.0017^†^	0.8043 ± 0.1348^†^
*R* ^2^	0.503	0.516	0.531

^†^ *p* < 0.01.

In the *cohort model* (Table 2 middle pane) there was a secular improvement across cohorts in the average levels of episodic memory consistent with the kernel densities in Fig 1. Against the pre-Depression era cohort as the reference, the level coefficients for the Depression era cohort, the War cohort and the post-War cohort are all statistically and significantly larger. (Initial estimation did not suggest that the rate coefficient differs for any of the cohorts, so in the models presented here age was not interacted with cohorts.) Compared to the baseline model, in the cohort model there were marked changes to the two age coefficients which delayed the peak to a decade later at 64.3 years.

In the *joint model* which accounted for attrition (Table 2 right pane), compared to the cohort model, the peak age is found at an earlier age of 62.1. For covariates other than the age coefficients in the two models, they are broadly comparable though slightly smaller in the joint model. In the joint model these cohort coefficients trace a step-by-step increase, so the cohort members’ levels of episodic memory are higher by 0.8, 1.6 and 2.5 words respectively. It seems to be a general observation that the coefficients given by the two models are similar with some attenuation in the joint model accounting for attrition.

Apart from the age and cohort coefficients, other covariates can also be compared and found to be stable across these three panes, giving confidence in the analysis. Focusing on demographic and social determinants in the joint model, the analysis found that women have higher levels of episodic memory compared to men, but couples are no different from non-couples in their episodic memory levels [16, 20].

Compared to the routine-manual class, the intermediate class and the managerial class have higher levels of episodic memory in a dose-response pattern. Following from this result on occupation, education attainment, particularly having some college education, also confers higher levels of episodic memory. Similarly, levels of wealth showed graded associations with episodic memory levels (people at the middle and top thirds of the wealth distribution have significantly higher levels of episodic memory by 0.1992 and 0.3748 respectively). Ethnic difference in episodic memory is also found with the minority reporting significantly lower levels. But this may be to do with the fact that the test instruments were in English instead of in the respondent’s native language. Social connections or structural social capital measured by whether in touch with friends in person, by letter, phone or email, are positively associated with levels of episodic memory (coefficient 0.0977, *p* < 0.001).

Physical problems and chronic conditions gave estimates that confirm and counfound expectations, ones of negative associations with episodic memory. Physical problems (a sum of problems with activities of daily living and instrumental activities of daily living and gross as well as fine muscle function and mobility) are negatively associated with episodic memory (coefficient −0.0435, *p* = 0.006), and so is suffering from diabetes (coefficient −0.2168, *p* = 0.038). But suffering from arthritis is associated with higher levels of episodic memory (coefficient 0.2088, *p* = 0.040). This nonetheless is known in the empirical literature and has been attributed to low level chronic inflammation which is associated with better cognition [50–52].

Behavioural risk factors gave expected estimates for this British sample, with a smoking coefficient that is negative though not significant [23] and regular drinking that is positive and significant [49]. Compared to no or mild physical exercise, exercise at moderate and vigorous levels were significantly associated with higher levels of episodic memory. A measure of depression or mood is included as control [16, 17], while an indicator of practice (whether had episodic memory tested before) also gave a positive coefficient (0.5859, *p* < 0.001).

## Discussion and Conclusion

This paper set out to trace a decade of cognitive ageing using a national sample of older Britons by estimating their individual and general trajectories of episodic memory. The general trajectories are found to take the shape of a curvilinear trajectory, not a steady or linear decline beyond the age of 50 years, consistent with an integrated view of cognitive development and cognitive ageing [40]. This shape is evidence in support of the first hypothesis of curvilinear trajectories of cognitive ageing.

Individual trajectories are found to vary around the general trajectory in their starting levels and their rates of change, replicating findings from studies of older volunteers in Manchester and Newcastle [14, 15] while extending them to the national population.

The data also revealed evidence in support of the second hypothesis relating to the Flynn effect. In addition, as mentioned above when presenting the results from the joint model, the final coefficients are generally attenuated, lending evidence in support of the third hypothesis regarding distortion when attrition is ignored.

**Fig 3:**
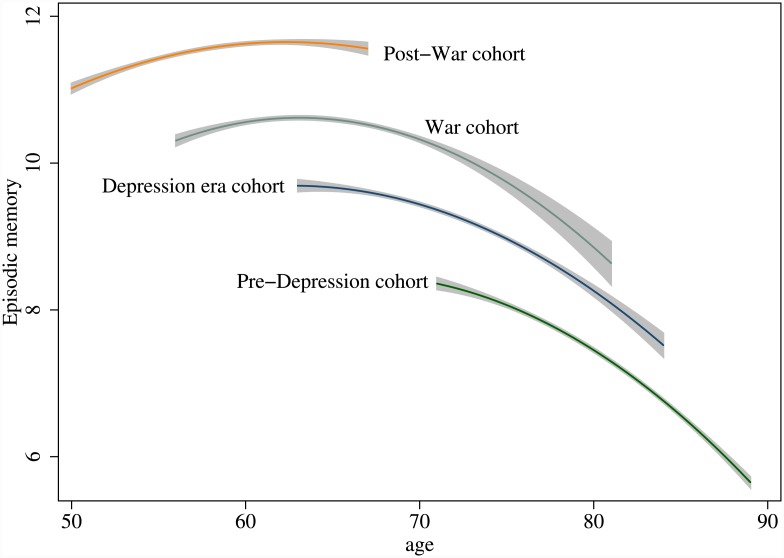
The curvilinear trajectories of episodic memory and cohort effects in later lives of older Britons. (Source: ELSA 2002–2013).

To summarise evidence in support of these hypotheses, plots of the predicted episodic memory for the four cohorts are given in Fig 3. These plots show for the first time the cohort effects and non-linear changes in episodic memory as older Britons age. The upward shifts in these four cohort trajectories are what lie behind the right-ward shifts in the four empirical densities shown above in Fig 1. Both the univariate empirical densities and the multivariate or joint model plots reinforce the evidence of cohort effects and non-linear changes in cognitive ageing in older Britons.

This paper is limited in some ways. On the dependent variable side, episodic memory does not exhaust all the cognitive abilities of older people, nor does it define cognitive ageing. Moreover other cognitive abilities change at different rates with advancing age [40]. Nonetheless episodic memory has been implicated in many important financial decisions in later life [10] and it deserves the focus it received here. It is also limited since it ignores measurement variance; it asssumes that the same construct is indicated by the same episodic memory scores over time, as in most other studies of cognitive ageing [13–15].

On the covariate side, social connections have been found here to be beneficial for cognitive functioning and they include both online and traditional connections (by phone or face to face meeting). Unfortunately, not many details of these online and traditional social connections were collected, preventing deeper investigations about the content passing through these myriad connections: whether it be merely instrumental (e.g. information about the recent government bond exclusively for pensioner) or emotional or aspirational.

The analysis found change in cognitive functioning to be non-linear instead of a stable decline in later life. Other studies tested only linear age coefficient [22]; ours tested linear and non-linear age coefficients as informed by an integrated theory of cognitive development and cognitive ageing [40] and by previous empirical work [14]. When so done, cognitive function is found to allow for an improvement in episodic memory well beyond the fifties. In contrast, in the US Health and Retirement Study for instance, Americans aged 51 years and older showed linear decline in episodic memory with advancing age [13]. It is possible that ignoring attrition, which is often substantial in any longitudinal ageing study, is partly responsible for this difference in the shapes of cognitive ageing trajectory found on different sides of the Atlantic. In any case, a comparison between the two countries is an obvious next step.

The possibility of maintenance of cognitive functioning in older people resonates with the literature on cognitive plasticity [53]. Animal studies of primates and non-primates have furnished evidence of replenishment of neurons even in later life, when the animals were given treatment after a particular shock to the brain caused limited damange. This rebounding or plasticity is manifest in the form of continuity and change that is not dominated by decline but by maintenance of cognitive function well into later life [54]. Although in the public mind losing one’s memory is a normal experience of getting old, these results suggest that in later life, maintenance of episodic memory is a real possibility for some.

More robust trajectories are uncovered here because the models accounted for a wide range of explanations and for attrition. For example without accounting for these chronic conditions one can attribute the cohort differences to the differences in the prevalence of chronic conditions. Plausibly the earliest cohort, who became adults when medical technology and clinical management were decidedly less advanced than those available at the latter part of the twentieth century, may present a higher prevalence of chronic conditions. These differences in prevalence can be argued to be the real reason for the difference in the levels of cognitive function across cohorts. But since these chronic conditions are accounted for, and more generally since an extensive set of risk factors is considered here, the resulting trajectories are considerably more robust.

The robust cognitive trajectories traced for these older Britons afford three claims. First, recall the peak ages above, which are found to be beyond 60 [31]. They mean that a decade into this ELSA study, a large group of older Britons, more specifically those who were in their early 50s at the start of the study, has just reached the peak of their cognitive abilities. Coupled with the fact that ELSA is a nationally representative sample, this is a positive prospect that hitherto has not been highlighted.

Second, this early-50s sliver, and more generally those who were born after the second world war, have higher peak levels (by a few words). In particular the differences found across cohorts demonstrated that the Flynn effect is observable in older people and enjoyed by the more recent cohorts. This has one implication that has not been widely appreciated: the higher peaks for the more recent cohorts can delay them from hitting a low cognitive ‘deficits’.

**Fig 4:**
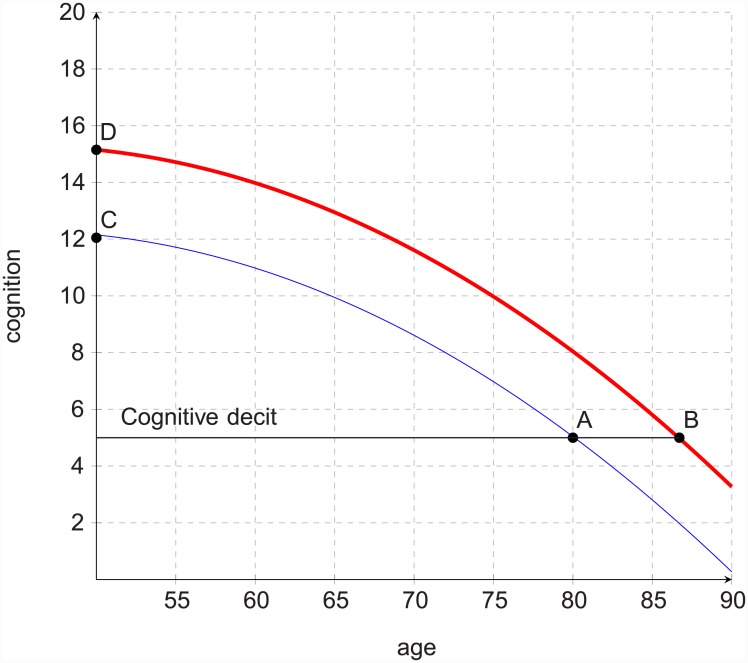
Delaying cognitive deficits, comparing early (blue) versus recent (red) cohorts.

We illustrate this cross-cohort delay in Fig 4. Because this idea of low cognitive deficit has not commanded general consensus [3, 55, 56], we refer to emerging studies in the literature which have proposed various deficit thresholds. Nordlund and co-authors, for instance, used the threshold of 1.5 standard deviations below clinical age norms to define mild cognitive deficits [57]; while Sotaniemi and co-authors used less than four delayed words recalled as the threshold for low cognitive deficits [58], and the investigators of the Assets and Health Dynamics of the Oldest Old study in the US used less than eight of the total mental scores as the threshold [59].

This illustration is not used to say that below the horizontal mark an individual is clinically impaired. It is used simply to say that it is possible to assess how long cohort members took to get from the peak level to the deficit mark, and subsequently to compare cohorts based on these times.

So do recent cohorts on average delayed meeting some deficit criteria? The evidence confirms. Since the more recent cohorts reached higher peak levels by a number of words, then with the same rates and accelerations as other cohorts, the recent ones will take longer to meet low cognitive deficits. Compare the time points *A* for an earlier cohort and *B* for a recent cohort in the illustration. It must be emphasised that this comparison illustrated not the specific claim that for the more recent cohorts the delay is a year or two, but the general claim that for them cognitive deficits may be delayed compared to for the earlier cohorts.

This illustration is useful for further research and policy. If and when further research has agreed or suggested that, say, the tenth percentile of the population norm is the threshold of deficits useful for public health policy, then the coefficients in Table 2 or at least the joint model proposed here can be used to work out the time difference between the most recent cohort and the earliest cohort. This exercise can be applied to episodic memory or other cognitive abilities [29, 40].

Thirdly, both the above claims are specimens of a positive view of cognitive ageing for the more recent cohorts. This view on ageing does not always hold sway. The well-being of older Americans as far as depression is concerned is a negative one [60]; likewise the well-being of the more recent cohorts of older Britons (ELSA) as far as frailty is concerned is a pessimistic one [61]. However another study has also found that, again using ELSA, their well-being measured in the domains of control, aspiration, self-realisation and pleasure, traced a more optimistic picture for the most recent cohort [27]. These seeming contradictions only serve to emphasise that even cognitive ageing, let alone ageing in general, speaks about a myriad of experience in later life. More research is needed to do justice to this experience, and given that the proportion of older people is set to increase, the response should not be delayed.

In conclusion the cognitive challenge identified above facing older Britons must be assessed against this finding that higher peak levels can mean postponement of cognitive deficits. Together with findings about amenable factors including physical activities and online social connections, such knowledge stands as a potential ally in responding to the challenge of the ageing population.

## Supporting Information

S1 Table (PDF)
