# Supplementary material for: Cognitive Ageing in Great Britain in the New Century: Cohort Differences in Episodic Memory
Source: PLoS One. 2015 Dec 29;10(12):e0144907. doi: 10.1371/journal.pone.0144907 (PMC4699214; doi:10.1371/journal.pone.0144907)
Supplement: S1 Table — (PDF) [file pone.0144907.s001.pdf]

Cognitive ageing in Great Britain in the new century: Supplementary Tables

Gindo Tampubolon[1]

1 University of Manchester

Table 1. Sensitivity analysis using decadal cohorts (ELSA 2002-2013)

Predictors	Episodic memory	
	Coeff.	Std. error
Constant	-6.9057	0.8725
Age	0.4097	0.0270†
Age^2	-0.0033	0.0002†
Cohort 60s	0.8093	0.1110†
Cohort 70s	1.6935	0.1235†
Cohort 80s	2.9422	0.1337†
Female	0.7232	0.0467†
Intermediate	0.9496	0.0578†
Managerial	1.1044	0.0547†
Married/cohab.	-0.0345	0.0435
College	0.9514	0.0542†
Middle tertile	0.1710	0.0370†
Top tertile	0.3484	0.0424†
Ethnic minority	-1.4015	0.1377†
Social connect.	0.0710	0.0184†
Physical problem	-0.0385	0.0062†
Hypertensives	-0.0754	0.0381
Diabetes	-0.1747	0.0624†
Cancer	0.0793	0.0637
CVD	0.0290	0.0473
Stroke	-0.6062	0.0889†
Arthritis	0.2173	0.0404†
Smoker	-0.0403	0.0435
Drink regularly	0.4743	0.0508†
Exercise mod.	0.2554	0.0348†
Exercise vig.	0.1043	0.0325†
CESD	-0.0770	0.0083†
Practice	0.6392	0.0722†
Within-person σ	0.8043	0.1348†
R^2	0.531	

† $p < 0.01$.
